# Detection of advanced brain aging in schizophrenia and its structural underpinning by using normative brain age metrics

**DOI:** 10.1016/j.nicl.2022.103003

**Published:** 2022-04-06

**Authors:** Chang-Le Chen, Tzung‐Jeng Hwang, Yu-Hung Tung, Li-Ying Yang, Yung-Chin Hsu, Chih‐Min Liu, Yi-Tin Lin, Ming-Hsien Hsieh, Chen-Chung Liu, Yi-Ling Chien, Hai‐Gwo Hwu, Wen-Yih Isaac Tseng

**Affiliations:** a Institute of Medical Device and Imaging, College of Medicine, National Taiwan University, Taipei, Taiwan; b Department of Bioengineering, University of Pittsburgh, Pittsburgh, PA, USA; c Department of Psychiatry, National Taiwan University Hospital, Taipei, Taiwan; d Department of Internal Medicine, National Taiwan University Hospital, Taipei, Taiwan; e AcroViz Inc., Taipei, Taiwan; f Department of Medical Imaging, National Taiwan University Hospital, Taipei, Taiwan; g Molecular Imaging Center, National Taiwan University, Taipei, Taiwan

**Keywords:** Brain age, Schizophrenia, Magnetic resonance imaging, Multimodality, Normative model, Machine learning

## Abstract

Conceptualizing mental disorders as deviations from normative functioning provides a statistical perspective for understanding the individual heterogeneity underlying psychiatric disorders. To broaden the understanding of the idiosyncrasy of brain aging in schizophrenia, we introduced an imaging-derived brain age paradigm combined with normative modeling as novel brain age metrics. We constructed brain age models based on GM, WM, and their combination (multimodality) features of 482 normal participants. The normalized predicted age difference (nPAD) was estimated in 147 individuals with schizophrenia and their 130 demographically matched controls through normative models of brain age metrics and compared between the groups. Regression analyses were also performed to investigate the associations of nPAD with illness duration, onset age, symptom severity, and intelligence quotient. Finally, regional contributions to advanced brain aging in schizophrenia were investigated. The results showed that the individuals exhibited significantly higher nPAD (P < 0.001), indicating advanced normative brain age than the normal controls in GM, WM, and multimodality models. The nPAD measure based on WM was positively associated with the negative symptom score (P = 0.009), and negatively associated with the intelligence quotient (P = 0.039) and onset age (P = 0.006). The imaging features that contributed to nPAD mostly involved the prefrontal, temporal, and parietal lobes, especially the precuneus and uncinate fasciculus. This study demonstrates that normative brain age metrics could detect advanced brain aging and associated clinical and neuroanatomical features in schizophrenia. The proposed nPAD measures may be useful to investigate aberrant brain aging in mental disorders and their brain-phenotype relationships.

## Highlights

• Novel metrics are proposed using the brain age paradigm with normative modeling.
• Normative brain age is validated to reveal advanced aging in schizophrenia.
• Men with schizophrenia have older brain age than women with the disorder.
• The brain age in white matter is positively associated with the negative symptom.
• The precuneus and uncinate fasciculus are markedly related to the advanced aging.

## 1 Introduction

Schizophrenia (SZ) undergoes neurobiological alterations that are involved in both neurodevelopmental and neurodegenerative processes (Kochunov and Hong, 2014, Pasternak et al., 2012, Rapoport et al., 2012) and manifests various impairments in brain structure and function (Brugger and Howes, 2017, Lawrie et al., 2008, Padmanabhan et al., 2015). Neuroimaging studies reported pronounced cerebral gray matter (GM) volume loss (Torres et al., 2016, Vita et al., 2012) and cortical thickness reduction primarily in the frontal and temporal areas (van Haren et al., 2011), resembling the changes in normal aging (Lemaitre et al., 2012). Diffusion magnetic resonance imaging (MRI) constantly reported altered mesostructure of white matter (WM) in SZ, which reflects the disconnection between cortical areas (Chen et al., 2018, Huang et al., 2018, Kelly et al., 2018, Wu et al., 2015a) and may result in cognitive impairments (Nazeri et al., 2013). *Voineskos et al.* reported that WM mesostructure was reduced in individuals with SZ, and the reduction pattern was similar to that in relatively older normal controls (NC) (Voineskos et al., 2010). These findings imply that individuals with SZ might potentially have an older brain.

A neuroimaging-based brain age paradigm has been widely used to investigate aberrant brain aging in neurological diseases and psychiatric disorders (Chen et al., 2019, Cole and Franke, 2017, Kaufmann et al., 2019, Koutsouleris et al., 2014). Using modern machine learning techniques, brain scans are transformed from high-dimensional neuroimaging features to a concise brain age marker. The established model can predict other individuals’ brain age. The predicted age difference (PAD), defined as the difference between an individual’s brain age and chronological age, is commonly used to indicate the brain aging status (Cole et al., 2015). Depending on the modality of neuroimaging data, the derived PAD reflects modality-specific brain aging. In individuals with SZ, GM-based brain age measures have demonstrated apparent brain aging in both early and chronic stages (Hajek et al., 2019, Koutsouleris et al., 2014, Nenadic et al., 2017, Schnack et al., 2016). It was reported that accelerated brain aging occurred in the early stage of the disease course (Schnack et al., 2016). Also, the extent of advanced brain age might be correlated with polygenic risk for SZ (Teeuw et al., 2021). In addition, advanced WM aging has been observed in SZ by using diffusion MRI techniques (Tonnesen et al., 2020, Wang et al., 2021). Overall, these findings suggest that advanced brain aging exists in individuals with SZ compared to the normal (Ballester et al., 2021, Koutsouleris et al., 2014, Nenadić et al., 2017), but the degree of advanced brain aging is heterogeneous and may vary with clinical outcomes such as symptom severity and cognitive deficit (Koutsouleris et al., 2014, Schnack et al., 2016, Wang et al., 2021).

Recent research has demonstrated that conceptualizing mental disorders as deviations from normative functioning illustrates a new perspective to investigate the heterogeneous neurobiology underlying psychiatric disorders at an individual level (Lv et al., 2021, Marquand et al., 2019, Wolfers et al., 2018). The normative modeling applied to neuroimaging features of a large-scale cognitively normal population-based cohort defines a normative range of neurobiological idiosyncrasies such as GM volume and WM mesostructure, providing personalized statistical inferences and being useful for parsing the heterogeneity in clinical cohorts. This approach may also offer a new viewpoint when investigating the aberrant brain aging in SZ. Hence, in this study, we leveraged the notion of normative modeling and applied it to the brain age paradigm, attempting to devise a more generalizable brain age metric for the investigation of brain aging in SZ on an individual basis. We introduced a brain age measure named “normalized PAD” (nPAD); nPAD was defined as a normalized difference between an individual’s and his/her demographic-matched peers’ brain age. By definition, nPAD indicates the deviation of an individual’s brain age from what is defined in the reference cohort, while conventional PAD is defined as the difference between one’s predicted age and chronological age. It is intuitive to quantify the extent of brain aging, but it lacks an objective reference when the inference is interpreted on an individual basis. For example, PAD may not be equivalent across the lifespan; the same amount of increased PAD at different stages of age might not be biologically identical. Furthermore, PAD directly derived from brain age models has an intrinsic statistical bias (Smith et al., 2019); that is, the PAD is correlated with chronological age. This hinders the unbiased estimation of correlation between PAD measures and age-related variables of interest such as duration of illness. Although some correction methods have been proposed to remove the bias based on a regression adjustment of the PAD (Beheshti et al., 2019, de Lange and Cole, 2020), these methods are prone to artificially inflate the model accuracy and have inherent circularity of age and age prediction, leading to over- or underestimated results. (Butler et al., 2021). The framework of nPAD might offer a solution to these limitations; nPAD is theoretically free of age-related bias in terms of its definition because the reference of comparison of one’s brain age is the peers’ brain age. Therefore, a fair comparison between different modalities or different cohorts can be made, and the correlation of brain age measures with age-related clinical variables can be estimated more reliably.

To capture a more comprehensive picture of brain aging in SZ from the standpoint of normative models, we constructed three brain age models based on the neuroimaging features of GM, WM, and their combination (i.e., multimodality) from two imaging modalities, and the subsequent normative models of brain age were established for the estimation of nPAD. We compared nPAD between individuals with SZ and NC. In addition, the clinical significance of advanced brain aging in SZ was investigated to explore the associations of nPAD with various phenotypes, including illness duration, onset age, symptom severity, and general cognition (i.e., full-scale intelligence quotient, FSIQ). Lastly, we investigated the GM and WM features that uniquely contributed to the advanced brain aging in SZ by testing the effect of group-by-feature interaction on nPAD. The identified key features allowed us to understand the structural underpinnings of aberrant brain aging in SZ.

## 2 Materials and methods

### 2.1 Participants

Individuals with SZ (N = 147; mean age = 31.1; standard deviation [SD] = 8.3; range 16–62; sex: 46.3% men; education: 14.3 [2.5] years) were consecutively recruited from the outpatient clinic of the Department of Psychiatry of National Taiwan University Hospital (NTUH). Individuals with SZ were diagnosed based on symptoms and clinical presentations which met the criteria of the Diagnostic and Statistical Manual of Mental Disorders‐5 (DSM-5). Diagnoses of SZ were made after comprehensive chart reviews and personal interviews performed by the experienced psychiatrists listed in the author byline. Individuals were excluded if they had schizoaffective disorder, bipolar disorder, substance abuse, intellectual disability, major systemic disease, or neurological diseases. Baseline symptoms were assessed using the Positive and Negative Syndrome Scale (PANSS), and FSIQ was measured using the Wechsler Adult Intelligence Scale—Third Edition (Chen et al., 2008, Kaufman and Lichtenberger, 2005). We also enrolled NC (N = 130; mean age = 30.8; SD = 8.5; range 16–62; sex: 48.5% men; education: 15.9 [1.2] years) who met the following inclusion criteria: MMSE score of ≥ 25 and none of the following: self-reported substance abuse, apparent brain injury and surgery, current serious health problems, and history of diagnosed neurological diseases or psychiatric disorders.

To construct brain age prediction models, we obtained brain images of 482 cognitively normal individuals (mean age = 36.9, SD = 19.1, range = 14–92; sex: 53.1% women) from the NTUH image database (Chen et al., 2020), including T1-weighted images and diffusion spectrum imaging (DSI) datasets, as the training set. Another independent set of 70 cognitively normal individuals (mean age = 36.8, SD = 19.9, range = 14–83; sex: 52.2% women) from the database was used to test the reproducibility of the brain age models. All 552 cognitively normal participants met the aforementioned inclusion criteria for NC (For detailed information, please refer to Supplementary Material S1.1). The Institutional Review Board of NTUH approved the study, and all participants provided written informed consent.

### 2.2 MRI image acquisition

All brain images used in this study were acquired on the same 3-Tesla MRI scanner (Tim Trio; Siemens, Erlangen, Germany) with a 32-channel phased-array head coil at the National Taiwan University Hospital. High-resolution T1-weighted imaging was performed using a three-dimensional magnetization-prepared rapid gradient-echo sequence with the isotropic spatial resolution of 1 mm^3. DSI was performed using a pulsed-gradient spin-echo echo-planar imaging sequence with a twice-refocused balanced echo that reduced distortions induced by the eddy current (Reese et al., 2003); the imaging parameters were *b*_max_ = 4000 s/mm^2 and in-plane spatial resolution = 2.5 mm^2. The diffusion-encoding acquisition scheme comprised 102 diffusion-encoding gradients corresponding to the Cartesian grids in the half-sphere of the three-dimensional diffusion-encoding space (Kuo et al., 2008). Each MRI scanning session was completed within 20 min. The details of imaging parameters are provided in Supplementary Material S2.1.

### 2.3 Image analysis

**Fig. 1:**
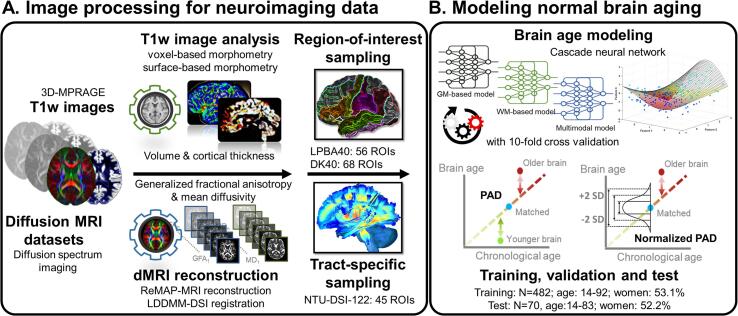
Processing pipeline and conceptual explanation of brain age. Subplot A illustrates the imaging processing for the T1-weighted images and diffusion spectrum imaging datasets. Subplot B represents the brain age models established using the data sampled from a normal population. Abbreviations: DK40 = Desikan-Killiany atlas; dMRI = diffusion MRI; DSI = diffusion spectrum imaging; GFA = generalized fractional anisotropy; GM = gray matter; LDDMM-DSI = large deformation diffeomorphic metric mapping for DSI; LPBA40 = LONI probabilistic brain atlas; MD = mean diffusivity; MPRAGE = magnetization prepared rapid gradient echo; MRI = magnetic resonance imaging; NTU-DSI-122 = National Taiwan University DSI template; PAD = predicted age difference; ReMAP-MRI = regularized mean apparent propagator MRI; ROI = region of interest; SD = standard deviation; T1w = T1-weighted; WM = white matter.

Before image data analysis, all T1-weighted images and DSI data underwent quality assurance procedures, detailed in Supplementary Materials S2.2. All structural and diffusion MRI datasets used in this study had satisfactory image quality. To extract GM features from the T1-weighted images, voxel-based morphometry and surface-based morphometry were performed using Computational Anatomy Toolbox (CAT12) (Gaser and Dahnke, 2016), an extension of Statistical Parametric Mapping 12 (Ashburner et al., 2014) (Fig. 1A). Voxel-based morphometry was applied to estimate voxel-wise regional volume features according to the LONI probabilistic brain atlas, which contains 56 regions of interest (ROIs) (Shattuck et al., 2008). Surface-based morphometry was employed to measure cortical thickness through projection-based thickness estimation (Dahnke et al., 2012). The estimated thickness features were sampled according to the 68 cortical ROIs included in the Desikan–Killiany atlas (Desikan et al., 2006). A total of 56 volumetric and 68 cortical thickness features were used to estimate the GM-based brain age. Please see Supplementary Materials S2.3 for details of the image processing.

WM features were extracted from DSI datasets using an in-house analytic pipeline to transform DSI data into tract-specific features (Chen et al., 2015) (Fig. 1A). The algorithm is detailed in Supplementary Materials S2.3. Briefly, diffusion indices, i.e., generalized fractional anisotropy (GFA) and mean diffusivity (MD), were reconstructed from DSI data using a regularized version of the mean apparent propagator MRI algorithm (Hsu and Tseng, 2018, Özarslan et al., 2013). A diffusion MRI registration algorithm (Hsu et al., 2012) was employed to minimize the variation of brain morphology across subjects. To sample tract-specific features, the resulting transformation maps were used to project the tract bundle coordinates predefined on the ICBM152 template in the MNI space to individuals’ diffusion index maps in the native space. The pipeline produced 45 tract-specific features for each index from each participant. Consequently, 45 GFA and 45 MD features were obtained to estimate WM-based brain age. The parcellation of GM and WM ROIs is detailed in Supplementary eTable.

### 2.4 Brain age modeling and nPAD calculation

The GM, WM, and multimodal brain age prediction models were constructed using the training set’s brain features, respectively (Fig. 1B). Sex was also included as a predictor. We adopted a 12-layer feedforward cascade neural network for the architecture of the brain age models (Chen et al., 2020) and used a 10-fold cross-validation procedure to assess model performance in the training phase. We then evaluated the reproducibility of the brain age models using an independent test set. Pearson’s correlation coefficient and mean absolute error (MAE) between predicted age and chronological age were calculated to quantify the model performance. Please see Supplementary Materials S1.2 for detailed descriptions of brain age modeling.

After the model performance was determined, we used the same training set to construct normative models to transform individuals’ brain-predicted age to nPAD scores (Fig. 1B). Gaussian process regression (GPR) (Rasmussen and Williams, 2004) was used to obtain regression estimates for the training set; the independent variables were chronological age and sex, and the dependent variable was brain-predicted age. The GPR model estimated the mean and SD of the training sample’s brain-predicted age at a certain age and sex. In the model inference phase, an individual’s brain-predicted age was transformed to nPAD by the formula:

$$nPAD=\;\frac{\mathrm{Predicted}\;\mathrm{Age}-{\hat{\overset{¯}{x}}}_{\mathrm{peers}}}{{\hat{S}}_{\mathrm{peers}}},$$

where ${\hat{\overset{¯}{x}}}_{\mathrm{peers}}$ and ${\hat{S}}_{\mathrm{peers}}$ were the estimated mean and SD of brain-predicted age of the peers with the same age and sex derived from the GPR normative model, respectively. The additional information on the estimation and validation of nPAD is provided in the Supplementary Materials S3. This normalization procedure is concordant with the notion of normative modeling (Marquand et al., 2016, Tung et al., 2021). Given that nPAD is a standardized value (i.e., Z-score), it is free of age-related bias (Smith et al., 2019) and remains the biological meaning of PAD. A higher value of PAD indicates that a person’s brain age is older than his/her *chronological age*; in contrast, a higher value of nPAD means that a person’s brain age is older than his/her *peers’ brain-predicted age* (Fig. 1B, 1C). As a benchmark, we used a well-established bias correction approach to calculate the “corrected PAD” (cPAD) (de Lange and Cole, 2020).

### 2.5 Statistical analysis

Three analyses were performed in the study. The first analysis was the comparison of nPAD between SZ and NC; the nPAD scores derived from GM, WM, and multimodal models were compared using Analysis of Covariance (ANCOVA), adjusting the education factor. The cPAD measures were also compared using ANCOVA while adjusting sex and education factors as a benchmark. Paired t-tests and Pearson’s correlation coefficients were employed to examine the difference and correlation, respectively, between nPAD-GM- and nPAD-WM scores in individuals with SZ and NC. In addition, within the SZ group, the comparison of sex difference in nPAD measures was investigated.

Multiple linear regression analysis was performed to assess the relationships between nPAD (as dependent variables) and clinical phenotypes (as independent variables). Three classes of clinical phenotypes were analyzed, namely symptom scores (i.e., PANSS positive, negative, and general scores), clinical factors (i.e., duration of illness, onset age, and antipsychotic dosage), and FSIQ. Three regression models were estimated for the three classes. In the FSIQ model, education was controlled.

To investigate regions that uniquely contributed to the advanced brain aging in SZ, we fitted multiple linear regression models to the nPAD measures. The dependent variable was nPAD (i.e. nPAD-GM or nPAD-WM), and the independent variables were an image feature (e.g., hippocampal volume), a group index (SZ and NC), an interaction term of the image feature with group index, and the covariates, including age, sex, and education. The regression model was built for each image feature. We tested the significance of the interaction term and calculated the effect size using Cohen’s *f^2^*. A significant interaction indicated that the relationship of the image feature with nPAD metrics was significantly distinct between SZ and NC, implying that this image feature was a candidate contributor to the aberrant brain aging in SZ. For all the analyses, the multiple comparison problem was addressed by Benjamini-Hochberg correction (Benjamini and Hochberg, 1995).

## 3 Results

### 3.1 Model performance and nPAD evaluation

**Fig. 2:**
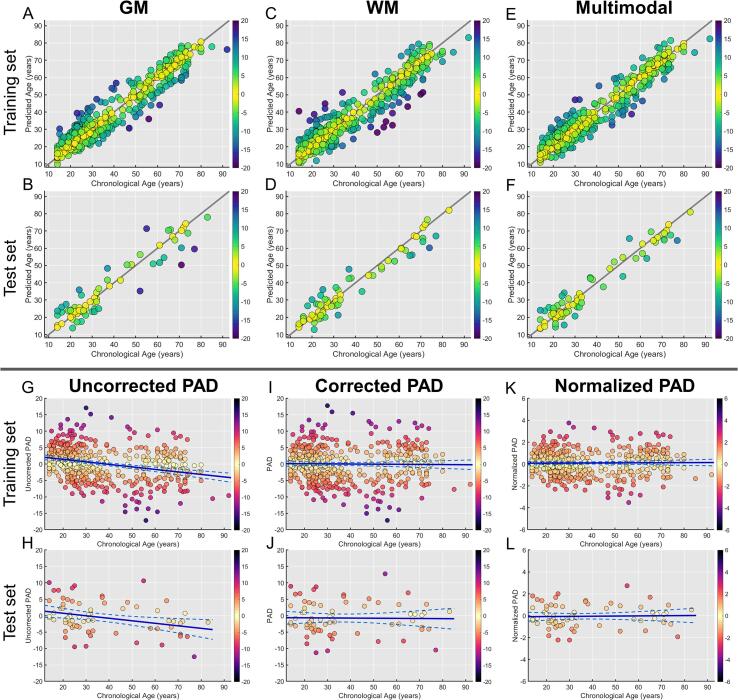
Scatter plots of the predicted age derived from gray matter (GM), white matter (WM), and multimodal brain age models against chronological age in the training set (A, C, E) and test set (B, D, F). Taking the multimodal-based brain age model for illustration, the scatter plots of the predicted age difference (PAD) metrics against chronological age are shown in the training set (G, I, K) and test set (H, J, L). Uncorrected PAD in both training and test sets is correlated with chronological age, indicating a significant age-related bias. In contrast, normalized PAD does not have the age-related bias, similar to corrected PAD.

We performed 10-fold cross-validation on the training set (N = 482), and the brain age models showed a strong linear correlation and low MAE between chronological age and predicted age based on GM (ρ = 0.956, MAE = 4.34), WM (ρ = 0.944, MAE = 4.76), and multimodal features (ρ = 0.964, MAE = 3.99). The models also accurately predicted brain age in the independent test set (N = 70) using GM (ρ = 0.943, MAE = 4.69), WM (ρ = 0.967, MAE = 3.95), and multimodal features (ρ = 0.969, MAE = 3.97) (Fig. 2A-F).

Correlation analysis of uncorrected PAD, cPAD, and nPAD against chronological age showed that nPAD was free of age-related bias, similar to cPAD. However, uncorrected PAD had a significant negative correlation with chronological age in training and test sets (Table 1 & Fig. 2G-L).

**Table 1:** Correlations of uncorrected PAD, cPAD, and nPAD with chronological age.

PAD metrics	GM	WM	Multimodal
Training set			
Uncorrected PAD	ρ = -0.342, P < 0.001	ρ = -0.358, P < 0.001	ρ = -0.287, P < 0.001
cPAD	ρ = -0.031, P = 0.495	ρ = -0.055, P = 0.226	ρ = -0.015, P = 0.742
nPAD	ρ = 0.026, P = 0.575	ρ = -0.016, P = 0.726	ρ = 0.013, P = 0.781
Test set			
Uncorrected PAD	ρ = -0.365, P = 0.002	ρ = -0.397, P = 0.001	ρ = -0.308, P = 0.009
cPAD	ρ = -0.096, P = 0.429	ρ = -0.006, P = 0.960	ρ = -0.017, P = 0.892
nPAD	ρ = -0.031, P = 0.800	ρ = 0.054, P = 0.656	ρ = 0.028, P = 0.819

Abbreviations: cPAD = corrected predicted age difference (PAD); nPAD = normalized PAD; GM = gray matter; WM = white matter.

### 3.2 Comparison results of nPAD

**Fig. 3:**
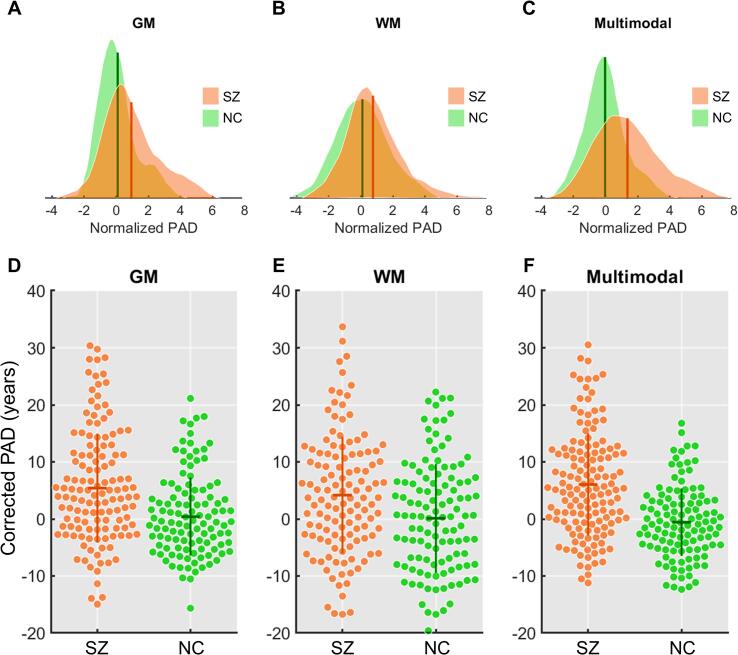
Distribution plots of normalized predicted age difference (A, B, C) and beeswarm plots of corrected predicted age difference (D, E, F) in schizophrenia (SZ) and normal controls (NC) based on different brain age models. The horizontal and vertical lines in the beeswarm dots indicate median and interquartile range, respectively. Abbreviations: PAD = predicted age difference; GM = gray matter; WM = white matter.

Table 2 summarizes the demographic characteristics of individuals with SZ and NC. ANCOVA revealed that all nPAD scores were significantly different between SZ and NC (Table 3, Fig. 3A-C), comparable with the statistical results obtained from cPAD metrics (Table 3, Fig. 3D-F). The results indicated that the individuals with SZ had a significantly advanced brain aging that was approximately 1 SD deviated from the population norm in terms of GM and WM features. This also suggested that the nPAD measures can achieve satisfactory sensitivity of detecting aberrant brain aging in SZ as analogous to that of cPAD metrics. Paired *t*-test showed that there was no significant difference between nPAD-GM and nPAD-WM in both SZ (*t*(146) = 1.03, P = 0.304) and NC (*t*(129) = -0.07, P = 0.948). Interestingly, a significantly positive correlation was found between nPAD-GM and nPAD-WM in SZ (ρ = 0.240, P = 0.004) but not in NC (ρ = -0.016, P = 0.858); these two correlation coefficients were statistically different (P = 0.033). Moreover, within the SZ group, we also compared the sex difference in nPAD measures. We found that nPAD-GM and nPAD-Multimodal in men with SZ (nPAD-GM: 1.402 [1.772]; nPAD-Multimodal: 1.957 [2.121]) were significantly greater (P = 0.001 & P = 0.004) than those in women with SZ (nPAD-GM: 0.540 [1.434]; nPAD-Multimodal: 0.843 [1.521]); however, there was no between-sex difference (P = 0.271) in nPAD-WM (men: 0.940 [1.917]; women: 0.614 [1.398]). In contrast, there was no sex difference in all nPAD measures in the NC group (nPAD-GM: men = 0.078 [1.126], women = 0.101 [1.195], P = 1.000; nPAD-WM: men = 0.072 [1.462], women = 0.128 [1.615], P = 0.919; nPAD-Multimodal: men = -0.111 [1.149], women = 0.055 [1.301], P = 1.000). Notably, the P-values shown here were corrected for multiple comparisons.

**Table 2:** Demographic characteristics of participants in each group.

Characteristics	Individuals with SZ	Normal Controls	P-values
N	147	130	–
Age (y)	31.1 (8.3)	30.8 (8.5)	0.767
Age range (y)	[16,62]	[16,62]	–
Sex (%)	46.3% men	48.5% men	0.714
Education (y)	14.3 (2.5)	15.9 (1.2)	<0.001
Age at onset (y)	23.4 (6.9)	–	–
Disease duration (y)	7.5 (7.0)	–	–
PANSS- positive score	13.1 (5.1)	–	–
PANSS- negative score	15.8 (7.2)	–	–
PANSS- general score	28.2 (8.4)	–	–
Daily antipsychotic dose, mean CPZ-equivalent (mg)	312.8 (269.8)	–	–
FSIQ score	93.8 (12.9)	–	–

Abbreviations: CPZ = chlorpromazine; FSIQ = full-scale intelligence quotient; PANSS = the Positive and Negative Syndrome Scale; SZ = schizophrenia.

**Table 3:** Comparisons of various PAD metrics based on different brain age models.

Brain age metrics	SZ (N = 147)	NC (N = 130)	*F*-values	Corrected P-values
nPAD-GM	0.939 (1.651)	0.090 (1.158)	*F*_(1,274)_ = 11.68	P = 0.002
nPAD-WM	0.765 (1.660)	0.101 (1.537)	*F*_(1,274)_ = 7.33	P = 0.007
nPAD-Multimodal	1.358 (1.900)	−0.027 (1.228)	*F*_(1,274)_ = 29.82	P < 0.001

cPAD-GM (years)	5.393 (9.56)	0.405 (6.84)	*F*_(1,273)_ = 12.33	P = 0.001
cPAD-WM (years)	4.178 (10.23)	0.113 (9.54)	*F*_(1,273)_ = 7.06	P = 0.008
cPAD-Multimodal (years)	6.030 (8.96)	−0.576 (5.92)	*F*_(1,273)_ = 31.16	P < 0.001

Abbreviations: SZ = schizophrenia; NC = normal controls; cPAD = corrected predicted age difference (PAD); nPAD = normalized PAD; GM = gray matter; WM = white matter.

### 3.3 Regression analysis of nPAD with phenotypes

To minimize the bias caused by outliers, we excluded individuals whose clinical factors and symptom scores exceeded three SDs (N = 10, 6.8% of the samples). In the regression model of nPAD with clinical factors, the age of onset exhibited a significant negative correlation with nPAD-WM. In contrast, the duration of illness and antipsychotic dose did not show significant associations with any of the nPAD scores (Table 4). In the regression model of nPAD with symptom scores, negative symptoms had significantly positive associations with nPAD-WM and nPAD-Multimodal (Table 4). In the regression model of nPAD with FSIQ, only nPAD-WM was significantly associated with FSIQ, while there was a marginal correlation between nPAD-Multimodal and FSIQ (Table 4).

**Table 4:** Regression models of nPAD with clinical factors, symptom scores, and full-scale intelligence quotient.

	nPAD-GM			nPAD-WM			nPAD-Multimodal		
	Estimate	SE	Corrected P-values	Estimate	SE	Corrected P-values	Estimate	SE	Corrected P-values
*Models for clinical factors*									
Duration of illness	0.0075	0.0225	1.000	−0.0252	0.0208	0.687	−0.0011	0.0262	0.967
Onset age	−0.0219	0.0250	0.383	**−0.0731**	**0.0232**	0.006*	−0.0463	0.0291	0.228
Antipsychotic dose	0.0007	0.0007	0.334	0.0010	0.0007	0.154	0.0012	0.0009	0.158
*Models for symptom severity*									
Positive	0.0145	0.0379	0.703	0.0100	0.0346	0.774	0.0384	0.0439	0.383
Negative	0.0327	0.0274	0.234	**0.0761**	**0.0250**	0.009*	**0.0741**	**0.0317**	0.042*
General	−0.0036	0.0254	0.886	−0.0109	0.0232	1.000	−0.0230	0.0294	1.000
*Models for full-scale intelligence quotient*									
Full-scale intelligence quotient	−0.0170	0.0132	0.203	**−0.0303**	**0.0119**	0.039*	−0.0308	0.0150	0.086

*: with statistical significance after adjusted by Benjamini-Hochberg correction.

Note: Ten individuals (6.8% of the samples) whose clinical factors and symptom scores exceeded 3 SDs were excluded.

Abbreviations: nPAD = normalized predicted age difference; GM = gray matter; WM = white matter; SE = standard error.

### 3.4 Regional impact on advanced brain aging in SZ

**Fig. 4:**
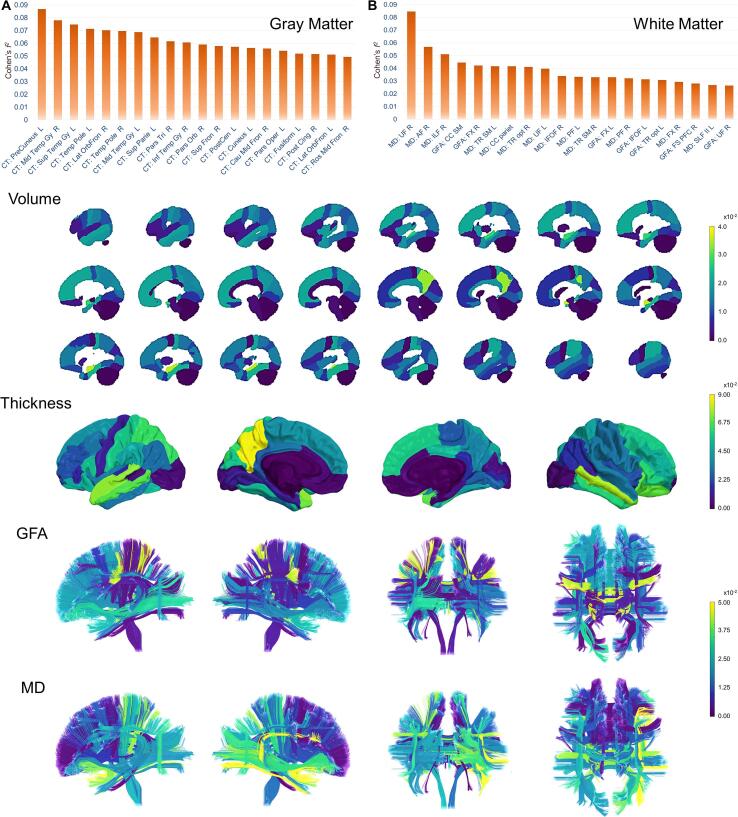
Bar charts of regional importance and illustration of corresponding brain maps in schizophrenia. The bar charts show the regions of the top 20 important features contributing to the normalized PAD scores of gray matter (A) and white matter (B). The color spectrum of effect size (Cohen’s *f*^2^) encodes the importance of feature contribution in the brain maps of volume, thickness, generalized fractional anisotropy (GFA), and mean diffusivity (MD). To best contrast the regional difference within a certain feature type, the color scale is different across feature types. For abbreviations of anatomical regions, please see Supplementary eTable.

To identify the regions that uniquely contributed to the increased nPAD scores in SZ, we fitted multiple linear regression to nPAD measures and tested the significance of the group-by-image-feature interaction for each image feature. A significant interaction denoted that the relationship of the image feature with nPAD metrics was significantly distinct between SZ and NC, implying that this image feature might have a significant impact on the contribution to the advanced brain aging in SZ. In the GM features, 24 image features were identified to have significant interactions. The features included cortical thickness in the bilateral precunei, middle temporal gyri, temporal poles, lateral orbitofrontal gyri, superior parietal gyri, etc. (Table 5). Notably, all the identified GM features belonged to cortical thickness. In WM, the identified features included MD in the bilateral uncinate fasciculi, right arcuate fasciculus, right inferior longitudinal fasciculus, right fornix, left perpendicular fasciculus, and the corpus callosum to the parietal lobes, and GFA in the right frontostriatal circuit to the prefrontal cortex only (Table 5). To visualize the distribution of these distinct features, the effect size of the interaction term (i.e. Cohen’s *f*^2^) was color-coded in the corresponding brain regions (Fig. 4). One may notice that the order of the features shown in Fig. 4A & 4B is slightly different from that shown in Table 5. This is due to the fact that the ranking in Fig. 4 was merely based on the value of the effect size (i.e. Cohen’s *f*^2^), while the features listed in Table 5 were the features which showed significance after statistical testing and multiple comparison correction.

**Table 5:** The image features with significant interaction terms.

Measures	Anatomical regions	Cohen’s *f^2^*	Coefficients of interaction term	Corrected P-values
Gray Matter Features				
CT	L precuneus	0.086	−5.740	0.003
CT	R middle temporal gyrus	0.080	−3.966	0.008
CT	L superior temporal gyrus	0.075	−4.118	0.009
CT	L temporal pole	0.071	−1.962	0.007
CT	R lateral orbitofrontal gyrus	0.070	−3.105	0.020
CT	R temporal pole	0.069	−1.935	0.008
CT	L middle temporal gyrus	0.069	−3.275	0.018
CT	L superior parietal gyrus	0.065	−4.388	0.021
CT	R pars triangularis	0.061	−3.845	0.020
CT	R inferior temporal gyrus	0.061	−2.910	0.021
CT	R pars orbitalis	0.059	−2.444	0.025
CT	R superior frontal gyrus	0.057	−4.085	0.021
CT	L postcentral gyrus	0.057	−3.932	0.013
CT	L cuneus	0.056	−3.673	0.018
CT	R caudal middle frontal gyrus	0.056	−3.153	0.034
CT	L fusiform gyrus	0.054	−3.108	0.033
CT	L pars opercularis	0.053	−4.159	0.009
CT	R posterior cingulate gyrus	0.052	−3.817	0.031
CT	L lateral orbitofrontal gyrus	0.051	−3.150	0.028
CT	R rostral middle frontal gyrus	0.049	−3.998	0.032
CT	L supramarginal gyrus	0.048	−3.858	0.033
CT	R superior parietal gyrus	0.047	−4.040	0.037
CT	L pars orbitalis	0.046	−2.455	0.035
CT	R precuneus	0.036	−3.427	0.045
White Matter Features				
MD	R uncinate fasciculus	0.085	28.111	0.002
MD	R arcuate fasciculus	0.057	31.298	0.036
MD	R inferior longitudinal fasciculus	0.051	22.917	0.039
MD	corpus callosum to parietal lobes	0.042	7.026	0.047
MD	L uncinate fasciculus	0.040	17.717	0.048
MD	L perpendicular fasciculus	0.033	14.504	0.045
MD	R fornix	0.030	2.541	0.043
GFA	R frontostriatal circuit to prefrontal cortex	0.028	−21.814	0.047

Abbreviations: CT = cortical thickness; GFA = generalized fractional anisotropy; MD = mean diffusivity; L = left; R = right.

## 4 Discussion

To investigate brain aging in SZ, we applied normative modeling to the brain age paradigm and estimated a normative brain age metric nPAD, which highlighted an individual’s brain aging deviation from a population-based norm. In this study, we validated that the nPAD measures can attain satisfactory sensitivity of detecting aberrant brain aging in SZ comparable to the popular brain age metrics used in previous studies cPAD. The nPAD measures revealed multifaceted advanced brain aging in SZ. Particularly, nPAD-Multimodal best distinguished the individuals from normal controls, confirming the superior sensitivity of a multimodal image marker (Cole, 2020). Among the three measures, nPAD-WM revealed significant associations with the age of onset, negative symptom scores, and FSIQ. Moreover, GM and WM regions that distinctly contributed to advanced brain aging mainly involved specific prefrontal, temporal, and parietal areas, revealing the neuroanatomical underpinnings of brain aging in SZ.

Normative modeling of neuroimaging data is an emerging approach to quantifying biological idiosyncrasy at an individual level with respect to a reference norm (Marquand et al., 2019). This approach has been widely used in multiple fields, such as the normative growth charts in bone densitometry (Van der Sluis et al., 2002). Here, we constructed normative models for brain age measures by applying GPR to a large sample of normal participants (n = 482) and applied the model to individuals with SZ, examining where each individual was located in the normal continuum (Marquand et al., 2016). To our knowledge, this is the first study introducing normative modeling to the brain age paradigm. A regular PAD quantifies the difference between individuals’ brain-predicted age and their chronological age. However, the biological interpretation of PAD might vary at different stages of chronological age. For instance, PAD of 5 years at the age of 30 might be biologically different from that at 60. The biological inference of this type of brain age metrics should have a reference group to compare the distance between normal and abnormal status. In contrast, nPAD is a normalized difference of brain-predicted age between an individual and his/her matched peers, so it is interpreted as an observed deviation of brain age from a population norm. This provides an individualized quantification of brain age with self-explanatory meaning to define the abnormality of brain aging from a statistical perspective. Furthermore, nPAD is free from linear and non-linear age-related bias (Supplementary Material S3). This implies that the nPAD metrics can handle the bias caused by a more complicated scenario (e.g. high-dimensional data with multiple interactions). Hence, nPAD would be more robust than regular PAD when compared across cohorts, allowing us to study the association of brain age metrics with age-related variables such as duration of illness. Moreover, the normalization procedure harmonizes prediction errors across different brain age models so that nPAD can be directly compared across modalities more reasonably. Also, the normalization in the nPAD estimation incorporates the marginal errors derived from the normative model so that the heteroscedasticity in the uncorrected PAD can be mitigated. By introducing the normative model, the nPAD metric is arguably a more generalizable marker to represent an individual’s brain aging status. In the present study, we adopted the group comparison design merely to validate nPAD, and did not fully demonstrate the hypothetical superiority of nPAD. Besides the preliminary results shown in the present study, further research is required to validate the effectiveness of individual inference based on the metrics.

Regarding the investigation in SZ, we discovered that the men with SZ had apparently more advanced brain age compared to the women with SZ, particularly in GM features. It has been known that sex differences generally exist in SZ in terms of etiology, age of onset, symptoms, and brain structures (Abel et al., 2010, Falkenburg and Tracy, 2014, Li et al., 2016). Men with SZ tend to show an earlier age at onset and a higher propensity to negative symptoms (Falkenburg and Tracy, 2014, Li et al., 2016). Previous review studies summarized that smaller medial temporal volumes, superior temporal gyrus, Heschl's gyrus, prefrontal lobe, etc. were commonly observed in males with SZ, although findings were not wholly consistent (Abel et al., 2010, Mendrek and Mancini-Marïe, 2016). With respect to the sex difference in brain age, one study reported that men with SZ had relatively advanced GM brain age (PAD = 3.37 years) than women with SZ (PAD = 1.07 years) (Nenadić et al., 2017); however, another study claimed that there was no sex difference in GM brain age (Lee et al., 2021). Our results demonstrated that males with SZ had more advanced GM and multimodal brain age than females, supporting the sex difference of brain aging in SZ.

The brain age measures derived from various imaging modalities reflect different aspects of neurophysiological mechanisms of brain aging (Cole, 2020). We found that nPAD-GM was not correlated with nPAD-WM in NC, but it was correlated with nPAD-WM in SZ, implying that the disease-induced advanced brain aging processes in GM and WM might be correlated. Previous studies reported that impaired WM mesostructure was correlated with GM reduction in multiple cortical and subcortical areas in SZ, suggesting a potential covariation of neuropathology between GM and WM regions (Miyata et al., 2009). Nevertheless, further study addressing the biological correlation between modalities is warranted to confirm the relationship modulated by the disorder.

Our results of advanced GM aging replicate the previous findings which reported that GM brain age in SZ was 3 to 5 years older than that in normal controls (Koutsouleris et al., 2014, Nenadić et al., 2017, Schnack et al., 2016). Based on the nPAD estimation, the discrepancy of 5 years is approximately one SD above the mean of the reference population. Among the GM features, we identified 24 GM features that had distinct contributions to nPAD-GM in SZ, including the bilateral precunei, middle temporal gyri, temporal poles, lateral orbitofrontal gyri, and superior parietal gyri, etc. (Table 5). The identified features were all the cortical thickness measures, implying an important role of cortical thickness in driving GM aging in SZ. When referring to the normal relationship between neuroanatomical features and brain age, the results of the interaction terms indicate that the increase in GM brain age relates to reduced cortical thickness in SZ in these regions. Among them, the alteration of the left precuneus was most evident. The precuneus is involved in neuropsychological processes causing impaired social perception and poor insight which are known to be affected in SZ (Cooke et al., 2008). One study demonstrated that the effect of prominent susceptibility genes for SZ was related to the topological changes of the precuneus (Wei et al., 2015). In addition, reduced superior and middle temporal gyri in SZ were also reported in multiple studies (Van Erp et al., 2018, van Haren et al., 2011), especially in individuals with poor outcomes (van Haren et al., 2011). Similarly, the aberrant topological feature of the temporal pole was shown in a large-scale study in SZ (Van Erp et al., 2018), and this was corroborated by longitudinal findings showing abnormality at disease onset and progressive aberration afterward (Lee et al., 2016). Moreover, altered lateral orbitofrontal gyrus and pars opercularis were also noted in SZ (Walton et al., 2018). Taken together, the GM features that most distinctly contributed to advanced brain aging in SZ are consistent with the impaired cortical loci typically found in SZ.

Besides GM, we found advanced WM aging in SZ, consistent with the study discovering abnormal WM aging in SZ (Cetin-Karayumak et al., 2019). Notably, we identified 8 WM features that exhibited distinct contributions to brain aging in SZ. The features included MD in the bilateral uncinate fasciculi, right arcuate fasciculus, right inferior longitudinal fasciculus, right fornix, left perpendicular fasciculus, and the corpus callosum to the parietal lobes, and GFA in the right frontostriatal circuit to the prefrontal cortex (Table 5). The results of the interaction suggest that the increase in WM brain age corresponded to increased MD and decreased GFA in SZ in the identified tract bundles compared with normal behavior. Among them, the right uncinate fasciculus was the most significant contributor to advanced WM aging. Disruptions in structural connectivity between frontal and temporal lobes have been implicated in SZ symptoms, including impaired visual attention and verbal abstraction (Kubicki et al., 2002). Individuals with SZ showed impaired WM mesostructure in bilateral uncinate fasciculi, and the tract mesostructure was associated with sensorimotor dexterity, emotion, and clinical remission (Huang et al., 2018, Jung et al., 2020, Singh et al., 2016). Another tract bundle connecting the frontal and temporal lobes, the right arcuate fasciculus, was altered in individuals with SZ and their unaffected siblings (Wu et al., 2015b). Our findings indicate that the fronto-temporal tract bundles commonly reported in SZ are key contributors to aberrant WM aging. Besides, other long-range fibers such as the inferior longitudinal fasciculus and corpus callosum should also be considered as potential markers of WM aging in SZ.

Considered as a neurodevelopmental disorder, SZ often manifests before the full maturation of WM (Duchatel et al., 2019). We found that some of the affected tracts, such as the fornix and inferior longitudinal fasciculus, mature in early adulthood (Lebel et al., 2012); this coincides with the time of peak risk for SZ (Di Biase et al., 2020, Ellison-Wright and Bullmore, 2009). It has been hypothesized that developmental timing might confer increased susceptibility to disruption of particular tracts (Di Biase et al., 2020). A stall in WM maturation may trigger psychosis (Carletti et al., 2012, Kochunov and Hong, 2014), and this would lead to an observed onset-related decline in WM mesostructure (Carletti et al., 2012). In our results, nPAD-WM was negatively correlated with the onset age, indicating that earlier onset age corresponds to more advanced WM brain age, which suggests that the earlier the impact on brain maturation, the older the brain appears.

From the perspective of brain-behavior relationships, different symptom dimensions of SZ might manifest various impairments in certain structure dimensions. Previous studies reported that brain age based on GM features might be associated with negative symptoms (Koutsouleris et al., 2014), PANSS total score (Schnack et al., 2016), and global functioning (Schnack et al., 2016). In our results, nPAD-WM and nPAD-Multimodal were correlated with the negative symptom subscales. Significant impairment of WM mesostructure in SZ has been reported in widespread tract bundles involving the uncinate fasciculi (Kubicki et al., 2002), inferior longitudinal fasciculi (Clark et al., 2011), corpus callosum (Nakamura et al., 2012), and other fiber bundles (Bopp et al., 2017), and the impairment was found to be correlated with the severity of negative symptoms (Bijanki et al., 2015, Bopp et al., 2017) and cognitive deficits (Castro-de-Araujo et al., 2018, Liu et al., 2013). Consistently, our results indicate that advanced WM brain age in SZ implicates worse cognitive functioning and negative symptoms. However, it should be noted that different symptoms and cognitive impairment might arise or disappear in association with acute exacerbations in SZ and fluctuate in severity in chronic individuals (Bopp et al., 2017), leading to heterogeneity in biological correlation with brain structures. Regarding the time course of brain aging in SZ, a large-sample longitudinal study reported that aberrant brain aging in SZ progressed fast shortly after the disease onset within 5 years (Schnack et al., 2016), suggesting that accelerated brain aging in SZ existed. However, in a much longer scope, SZ might have relatively stable brain aging following acute psychosis (Schnack et al., 2016, Shahab et al., 2019), and this speculation is compatible with our null results of the association between illness duration and brain age.

The study has limitations. Our preliminary findings were derived from the cross-sectional design and await validation with a longitudinal study. Although no correlation was found between antipsychotic dosage and brain age indices, the medication effect might be overlooked by a brief estimate of the mean CPZ-equivalent (Fusar-Poli et al., 2013). Further research with more detailed medication records is warranted to investigate the medication effect on brain aging in SZ. Regarding the control for image artifacts such as susceptibility-induced distortion and motion artifact, although we adopted quality assurance procedures including prospective screening, retrospective analysis, and a two-step registration-based framework to ensure that the artifacts would not significantly affect the image quality, further study is warranted to investigate the impact of artifacts on brain age estimation.

In conclusion, we incorporate normative modeling into the brain age paradigm and introduce nPAD to detect advanced brain aging in SZ. Being free of age-related bias, nPAD allows us to explore the relationships between brain age and other age-related phenotypes more reliably. It also allows us to assess the status of brain aging across different models of modalities and different stages in the lifespan. Therefore, the proposed nPAD metrics may be a potential imaging marker for quantifying brain aging status, which is useful to investigate brain-phenotype relationships and reflect deviant trajectories of the brain in psychiatric or neurological diseases.

### CRediT authorship contribution statement

**Chang-Le Chen:** Conceptualization, Methodology, Software, Formal analysis, Investigation, Writing – original draft, Visualization. **Tzung‐Jeng Hwang:** Conceptualization, Supervision, Resources, Writing – review & editing. **Yu-Hung Tung:** Validation, Data curation. **Li-Ying Yang:** Validation, Data curation. **Yung-Chin Hsu:** Software, Validation. **Chih‐Min Liu:** Resources, Data curation. **Yi-Tin Lin:** Resources, Data curation. **Ming-Hsien Hsieh:** Resources, Data curation. **Chen-Chung Liu:** Resources, Data curation. **Yi-Ling Chien:** Resources, Data curation. **Hai‐Gwo Hwu:** Resources, Data curation. **Wen-Yih Isaac Tseng:** Conceptualization, Supervision, Writing – review & editing, Funding acquisition.

## Declaration of Competing Interest

The authors declare that they have no known competing financial interests or personal relationships that could have appeared to influence the work reported in this paper.
