# Supplementary material for: Detection of advanced brain aging in schizophrenia and its structural underpinning by using normative brain age metrics
Source: Neuroimage Clin. 2022 Apr 6;34:103003. doi: 10.1016/j.nicl.2022.103003 (PMC9018160; doi:10.1016/j.nicl.2022.103003)
Supplement: Supplementary data 1 [file mmc1.docx]

**Supplementary Materials**

S1 Brain Age Estimation

S2 Image Data Processing

S3 Estimation and Validation of Normalized Predicted Age Difference

References

**S1 Brain Age Estimation**

**S1.1 Participants**

The brain age models were created by using the neuroimaging data from National Taiwan University Hospital (NTUH) database. This database contained a training set (N = 482) and a test set (N = 70), which were used to establish brain age models and evaluate model performance, respectively (training set: mean age = 36.9 years, max = 92, min = 14, female proportion = 53.1%; test set: mean age = 36.8 years, max = 83, min = 14, female proportion = 52.2%). The distributions of age and sex in the 2 sets were statistically identical. The participants of the 2 sets were cognitively normal and met the recruitment criteria, including a Mini-Mental State Examination score of 25 or above, no self-reported substance abuse at least in the past 3 years, no history of significant brain injury or brain surgery, no current serious health problems such as congestive heart failure or cancer, and no history of significant neurological diseases or psychiatric disorders. Participants who did not meet the safety and health-related criteria for MRI scanning were excluded.

**S1.2 Establishment of Brain Age Prediction Models**

The details of the MRI imaging parameters and image processing are described in S2. After conducting image processing, the input data for gray matter (GM)-based brain age modeling used the volume and cortical thickness features in regions of interest (ROIs), whereas that for white matter (WM)-based brain age modeling used the tract-specific features of generalized fractional anisotropy (GFA) and mean diffusivity (MD). Consequently, the neuroimaging features of the GM- and WM-based model input consisted of 124 and 90 features, respectively. The sex factor was also included as a predictor in the models. Twelve-layer feed-forward cascade neural network models, which provide an accurate prediction with flexible model architecture for transfer learning, were used to predict age (4). The cascade neural network is a feed-forward neural network involving connections from the input and every previous layer to the subsequent layer. This network is similar to a simplified fully connected version of a dense block in densely connected convolutional networks, which avoid the vanishing-gradient problem and strengthen feature propagation (5). The loss function of model optimization was specified as a mean square error function, which was optimized using a gradient descent algorithm with an adaptive learning rate and constant momentum. A 10-fold cross-validation procedure was adopted within the training set to estimate brain age model performance. Validation set performance was used to stop the model parameter updates. The training procedure was implemented using MATLAB R2019a (MathWorks Inc., Natick, MA, USA) with an NVIDIA GeForce RTX 2080Ti (NVIDIA Inc., Santa Clara, CA, USA) graphics processing unit for accelerated computing. The performance of the trained brain age models was tested by predicting the brain age of individuals in the test set. To quantify model performance, Pearson’s correlation coefficient and mean absolute error between the predicted age and chronological age were calculated.

**S2 Image Data Processing**

**S2.1 MRI Imaging Parameters**

The neuroimaging data in the NTUH database were acquired using a 3T Siemens TIM Trio scanner with a 32-channel phased-array head coil, with the same imaging protocol used for all data collection. High-resolution T1-weighted imaging was performed using a 3D magnetization-prepared rapid gradient echo (3D-MPRAGE) sequence: repetition time/echo time (TR/TE) = 2000/3 ms, flip angle = 9°, field of view (FOV) = 256 × 192 × 208 mm^3^, and acquisition matrix = 256 × 192 × 208; this resulted in an isotropic spatial resolution of 1 mm^3^. The imaging protocol for diffusion-weighted images followed that designed for diffusion spectrum imaging (DSI). The DSI datasets were acquired using the diffusion pulsed-gradient spin-echo echo-planar imaging sequence with a twice-refocused balanced echo (1, 2): TR/TE = 9600/130 ms, slice thickness = 2.5 mm, acquisition matrix = 80 × 80, FOV = 200 × 200 mm^2^, and in-plane spatial resolution = 2.5 × 2.5 mm^2^. The diffusion-encoding acquisition scheme used in this dataset followed the DSI framework published previously (2), in which 102 diffusion-encoding gradients were applied corresponding to the Cartesian grids in the half sphere of the 3D diffusion-encoding space (*q*-space) within a radius of 3 units equivalent to b_max_ = 4000 s/mm^2^ (3). Because the *q*-space data were real and symmetrical around the origin, the acquired half-sphere data were projected to ﬁll the other half of the sphere.

**S2.2 Image Quality Assurance**

Before we performed data analysis, all T1-weighted images underwent quality assurance (QA) procedures which are included in the Computational Anatomy Toolbox 12 (CAT12; http://dbm.neuro.uni-jena.de/cat.html), a retrospective QA framework for empirical quantification of quality differences. Retrospective QA involved automatic evaluation of essential image qualities such as noise, inhomogeneity, and image resolution. These quality measures were scaled to a rating scale, and “good” image quality level was required. Additional visual inspection was conducted to examine whether artifacts, including severe motion and abnormal lesions, remained in the images. All diffusion datasets also underwent QA procedures, including examinations for the signal-to-noise ratio (SNR), degree of alignment between T1- and diffusion-weighted images, and the motion-induced signal dropout (6). The SNR was evaluated by calculating the mean signal of an object divided by the standard deviation (SD) of the background noise (7). In practice, the signal was determined using a central square of an image for each slice, and the noise was averaged from 4 corner regions. Diffusion datasets with an SNR higher than mean SNR minus 2.5 SDs at their site were included. The degree of within-subject alignment between T1- and diffusion-weighted images was evaluated by calculating the spatial correlation between the T1-weighted image–derived WM tissue probability map and the diffusion-weighted image–derived GFA map. Higher spatial correlation indicated greater spatial alignment between T1- and diffusion-weighted images. In addition, because of the relatively long scan time of DSI, in-scanner head motion would inevitably cause signal dropout in diffusion-weighted images, particularly in those with high *b* values. For this reason, all participants lay on the MRI table with the head packed with expandable foam cushions to restrict head movement. All acquired DSI datasets (5,712 images per participant) were examined by comparing the signal in the central square of each image with the predicted signal attenuation. Signal deviation from the predicted distribution was considered signal loss. Data with more than 60 images of signal dropout per participant (1% of the total diffusion-weighted images) were discarded. Notably, a prospective visual inspection was conducted to exclude datasets presenting severe head motion or unexpected brain lesions during scanning.

**S2.3 Image Feature Processing**

In the image feature processing for GM, voxel-based morphometry and surface-based morphometry were used to analyze the 3D MPRAGE data. The image analyses were performed using an extension of the Statistical Parametric Mapping package (SPM12; Wellcome Department of Imaging Neuroscience, London, UK; www.fil.ion.ucl.ac.uk/spm) (8) called CAT12. For voxel-based morphometry analysis, the structural imaging data were preprocessed using the default settings of the CAT12 toolbox, including corrections for bias-field inhomogeneity and segmentation into GM, WM, and cerebrospinal fluid, followed by spatial normalization to the ICBM template in MNI space (voxel size: 1.5 × 1.5 × 1.5  mm^3^) with SHOOT registration (9). The LONI probabilistic brain atlas, containing 56 ROIs, was used as a reference of volumetric tissue compartments (10) to estimate the volume of each ROI. For surface-based morphometry analysis of cortical thickness, we applied the automated surface-preprocessing algorithms included in the CAT12 toolbox that enable the estimation of cortical thickness of the left and right hemispheres by using the projection-based thickness method (11). Here, cortical thickness was determined by estimating the WM distance based on tissue segmentation. We used WM distance and a derived neighbor relationship to project local maxima (which is equal to the cortical thickness) onto other GM voxels. This approach included partial volume correction and correction for sulcal blurring and sulcal asymmetries. The Desikan–Killiany cortical atlas containing 68 cortical ROIs was employed to sample cortical features (12). In this manner, 56 volumetric features and 68 cortical thickness features were obtained to estimate GM-based brain age.

In the image processing for WM, we used an in-house algorithm called tract-based automatic analysis (13). First, the diffusion indices, including GFA and MD, derived from the diffusion MRI dataset were computed using the regularization version of the framework of mean apparent propagator (MAP)-MRI (14, 15). The signal in 3D diffusion-encoding space was fitted with a series expansion of Hermite basis functions, which describe diffusion in various microstructural geometries (16). The zero-order term in the expansion series contained the diffusion tensor that characterizes the Gaussian displacement distribution. Higher-order terms in the expansion series were the orthogonal corrections to the Gaussian approximation, and these were used for reconstructing the average propagator. The MD in each voxel was determined by calculating the mean of the 3 eigenvalues of the diffusion tensor (17, 18). We quantified GFA as the SD of the orientation distribution function divided by the root mean square of the orientation distribution function (19). To extract effective features of WM, the diffusion indices were sampled according to the spatial coordinates of 45 predefined major fiber tract bundles over the whole brain, which were constructed in the DSI template NTU-DSI-122 (20) through deterministic streamline-based tractography with multiple ROIs defined in the automated anatomical labeling atlas (21). In practice, the sampling coordinates were transformed from NTU-DSI-122 into individual DSI datasets with the corresponding deformation maps. The deformation maps were obtained through 2-step registration, which included anatomical information provided by the T1-weighted images (22) and microstructural information provided by the DSI datasets (23). The sampling coordinates were aligned with the proceeding direction of each fiber tract bundle, and diffusion indices were sampled in the native space along the sampling coordinates normalized and divided into 100 steps. Having sampled the diffusion indices, we averaged the indices across 100 steps along each tract bundle. Finally, 45 GFA features and 45 MD features were obtained for estimating WM-based brain age.

**S3 Estimation and Validation of Normalized Predicted Age Difference**

**S3.1 Estimation of Normalized Predicted Age Difference**

In this study, we proposed a new definition of brain age measure called “normalized PAD” (nPAD); nPAD was defined as a normalized difference between an individual’s and his/her demographic-matched peers’ brain age. By definition, nPAD indicates the deviation of an individual’s brain age from what is defined in the reference cohort. nPAD takes into account prediction errors of brain age models, so it is theoretically free of age-related bias (24). In practice, after a brain age prediction model was established by using the training set, we further used this set to construct a normative model to transform individuals’ brain-predicted age to nPAD scores. Here, we employed Gaussian process regression (GPR), a flexible non-linear machine learning approach for regression, to obtain regression estimates of the normative model; the independent variables were chronological age and sex, and the dependent variable was brain-predicted age. The GPR model estimated the mean and SD of the training sample’s brain-predicted age at a certain age and sex. In the model inference phase, an individual’s brain-predicted age was transformed to nPAD by the formula: $nPAD= \frac{Predicted Age - {\hat{\bar{x}}}_{peers}}{\hat{S}_{peers}}$, where ${\hat{\bar{x}}}_{peers}$ and $\hat{S}_{peers}$ were the estimated mean and SD of brain-predicted age of the peers with the same age and sex derived from the GPR normative model, respectively. The proposed nPAD is essentially similar to predicted age difference (PAD); a higher index indicates an older brain aging status, but nPAD represents the degree of brain aging from a statistical perspective. It provides an individualized quantification of brain age with respect to a normative model, so it is readily evident to define abnormality of brain aging.

**S3.2 Validation of Normalized Predicted Age Difference**

In this study, nPAD was devised to describe the status of an individual’s brain aging with reference to the normal distribution of age- and sex-matched peers, thus it can be viewed as a PAD equivalent that is free of age-related bias. As for the common metrics used for the evaluation of brain age prediction, such as mean absolute error (MAE) and Pearson’s correlation between predicted age and chronological age, we did not expect that nPAD would outperform other brain age measures such as uncorrected PAD or corrected PAD (cPAD); cPAD is a brain age metric devised to remove age-related bias by using linear fitting correction (25). Instead, we assessed the performance of nPAD based on its resistance to age-related bias, and compared the performance with that obtained from uncorrected PAD (with age-related bias) and cPAD (without linear age-related bias). Uncorrected PAD was simply defined as $PAD= Predicted age-chronological age$, and cPAD was defined as $cPAD= \frac{Predicted age - \beta}{\alpha}-chronological age$, where $\alpha$ and $\beta$ were the parameters estimated based on the training set according to the following formula: ${Predicted age}_{training}= \alpha\times{Chronological age}_{training}+\beta$. Here, we designed two experiments to validate the resistance of nPAD to the age-related bias. The first experiment used simulated data to test whether nPAD can remove the age-related bias linearly and non-linearly, and the second experiment adopted real data to evaluate the performance. The ideal PAD measures should have no significant correlations with chronological age.

In the first experiment, we simulated data for brain age prediction and the resulting nPAD estimation based on the real image features derived from the data in the Cam-CAN database (26). The Cam-CAN database provides a population-based cohort that includes 616 cognitively normal subjects whose age ranges from 18 to 88 years old. In this cohort, high-resolution T1-weighted images are available for the subjects. We extracted gray matter (GM) features from the T1-weighted images by using the same analytic method in the Materials and Methods. A total of 56 GM volumetric features were obtained as the real data for the experiment. We utilized Copulas (27) to simulate GM features. Copulas are functions that model dependencies among random variables, providing an approach to create distributions that describe correlated multivariate data. To economize the computation load, we modeled the relationship of chronological age with each of GM features and simulated the data based on the modeled distribution (Supplementary Figure 1). In this way, we generated 1000 simulated samples as a training set and additional 1000 simulated samples as a test set without adding unstructurized noise. The cascade neural network described in the study was used as the model architecture of brain age prediction. The simulated GM features were used to predict age in the training set, and the prediction model was then applied to the test set to estimate the brain-predicted age. After the brain-predicted age was estimated, uncorrected PAD, nPAD, and cPAD were calculated based on the abovementioned methods. To evaluate the performance of age-related bias elimination, the linear, quadratic, and cubic correlations were estimated for each PAD measure.


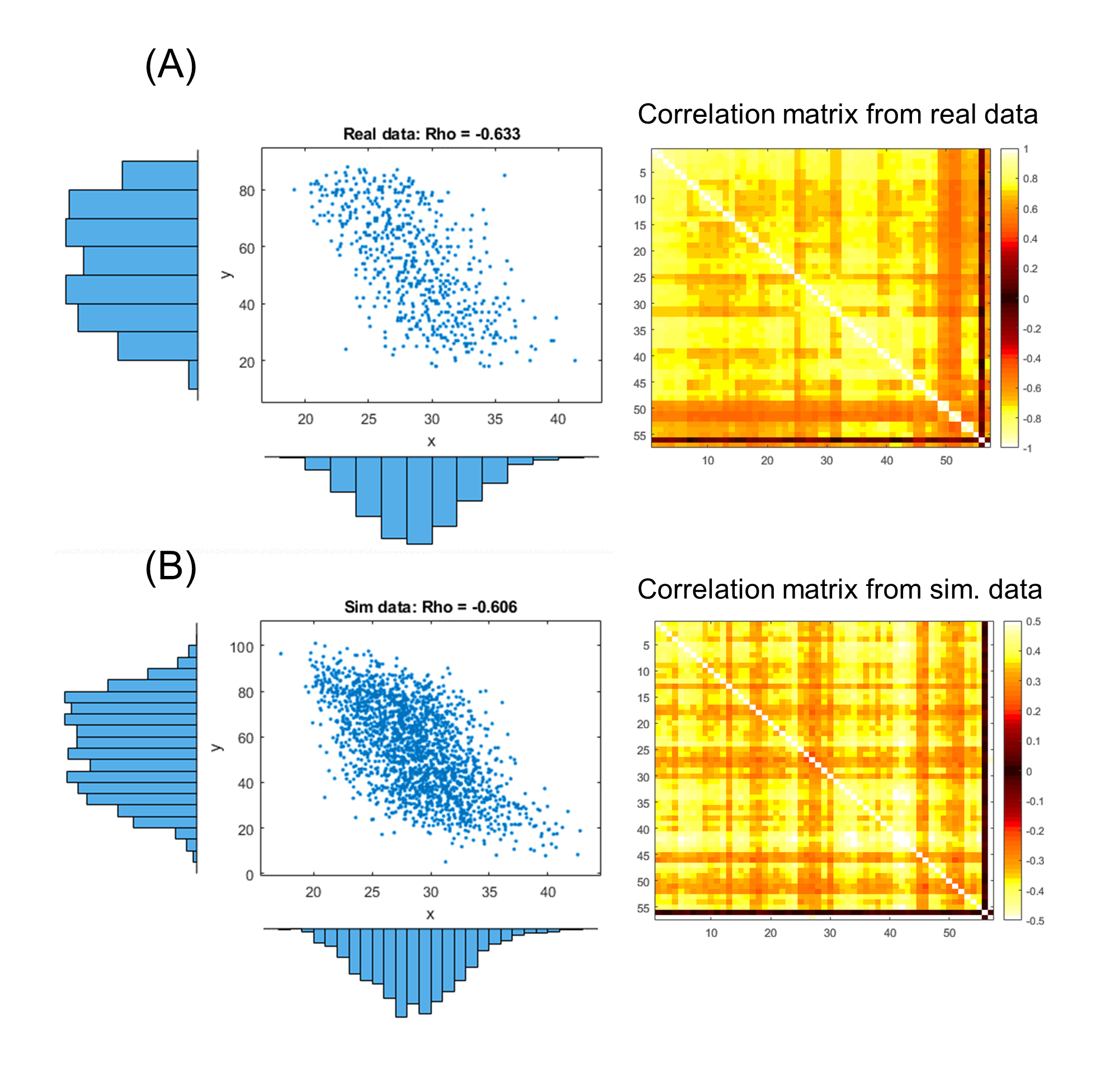


Supplementary Figure 1. The demonstration of simulated sample and the correlation between simulated gray matter (GM) features. The scatter plot in the subplot A shows the bivariate distribution between chronological age (marked as Y) and a GM feature (marked as X) based on the real data, and the correlation matrix next to the scatter plot displays the correlation between each real GM features. In the subplot B, the visualization of bivariate distribution and correlation matrix is based on the simulated GM features. Notably, due to the fact that only the relationships of chronological age with each of GM features are modeled, the correlations between GM features in the simulated data may be different from those in the real data.

The results of the first experiment (Supplementary Figure 2 & Supplementary Table 1) showed that there were significantly linear and non-linear correlations of uncorrected PAD with chronological age in both training and test sets, indicating that a significantly

age-related bias existed. In contrast, nPAD did not show any significant correlation with chronological age in a linear or non-linear manner, suggesting that nPAD was free of the bias. On the other hand, cPAD only removed the linear effect of the bias in both training ans test sets. This simulation study demonstrates that the estimation of nPAD is not contaminated by the age-related bias in either linear or non-linear manner.


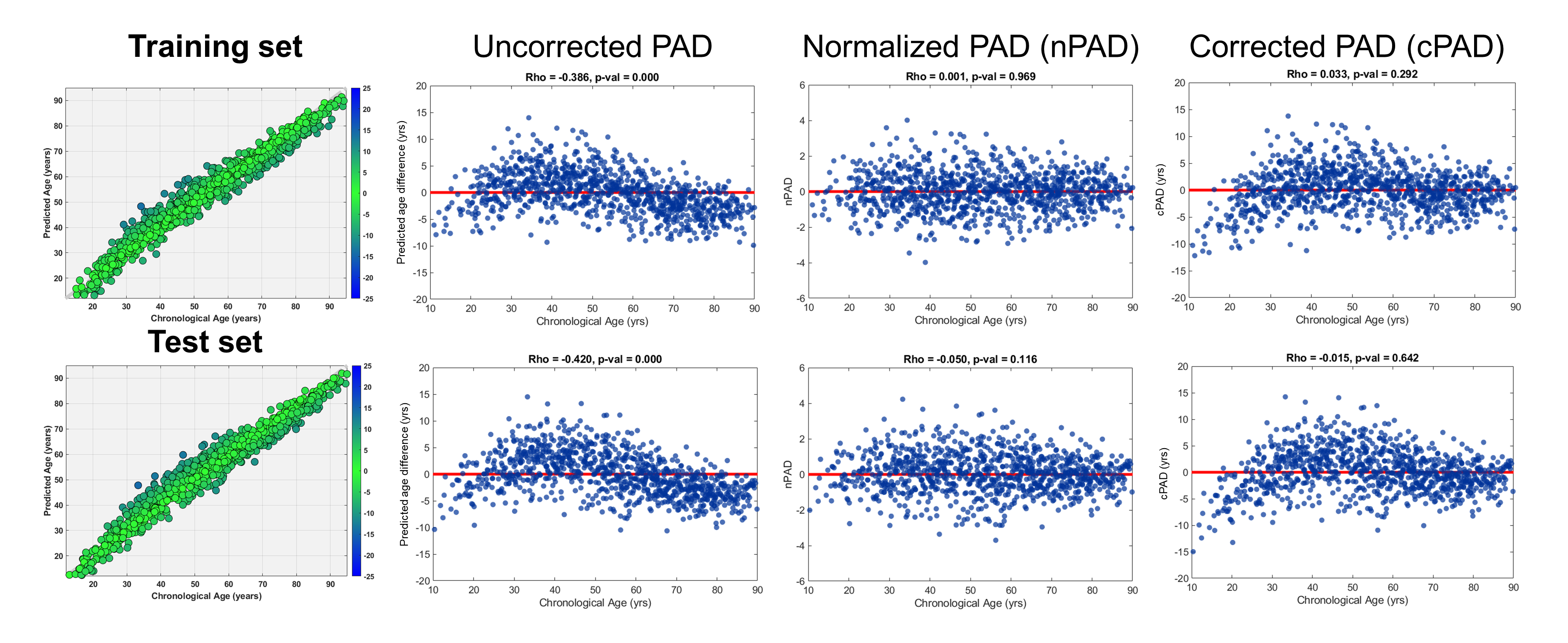


Supplementary Figure 2. The scatter plots of the PAD measures with chronological age. The results were derived from the simulated data.


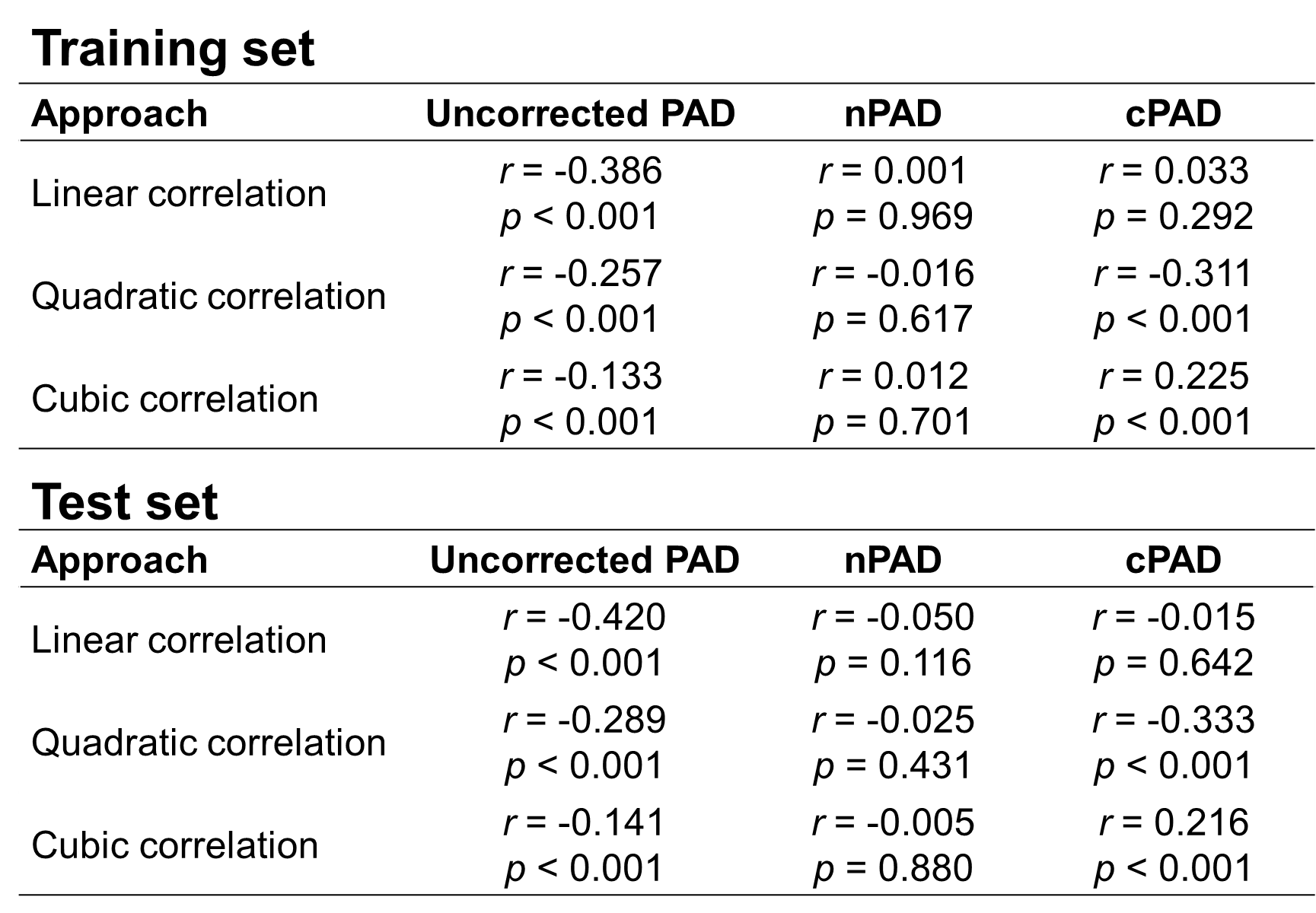


Supplementary Table 1. The linear and non-linear correlations of the PAD measures with chronological age. The results were derived from the simulated data.

The second experiment employed the same workflow in the first experiment; the only difference was that this time we used the real GM image features obtained from the population-based cohort to evaluate the performance. Firstly, we randomly split the entire dataset (N = 616) into the training (N = 493) and test set (N = 123) according to the 80/20 rule. Following the same workflow, we used 56 GM features to predict brain age and transformed it to the PAD measures. The results showed that there were significantly linear and non-linear correlations of uncorrected PAD with chronological age in both training and test sets. However, the nPAD did not show any significant correlations with chronological age in a linear or a non-linear manner, suggesting that nPAD was free of the bias. On the other hand, the cPAD only removed the linear effect of the bias in the training set, with the non-linear effect remained. In the test set, it seemed that cPAD can remove the non-linear effect of the bias, but it might be a false-negative result due to relatively sparse samples in the set. In summary, the results validated that nPAD metrics are not affected by the age-related bias in both linear and non-linear manner.


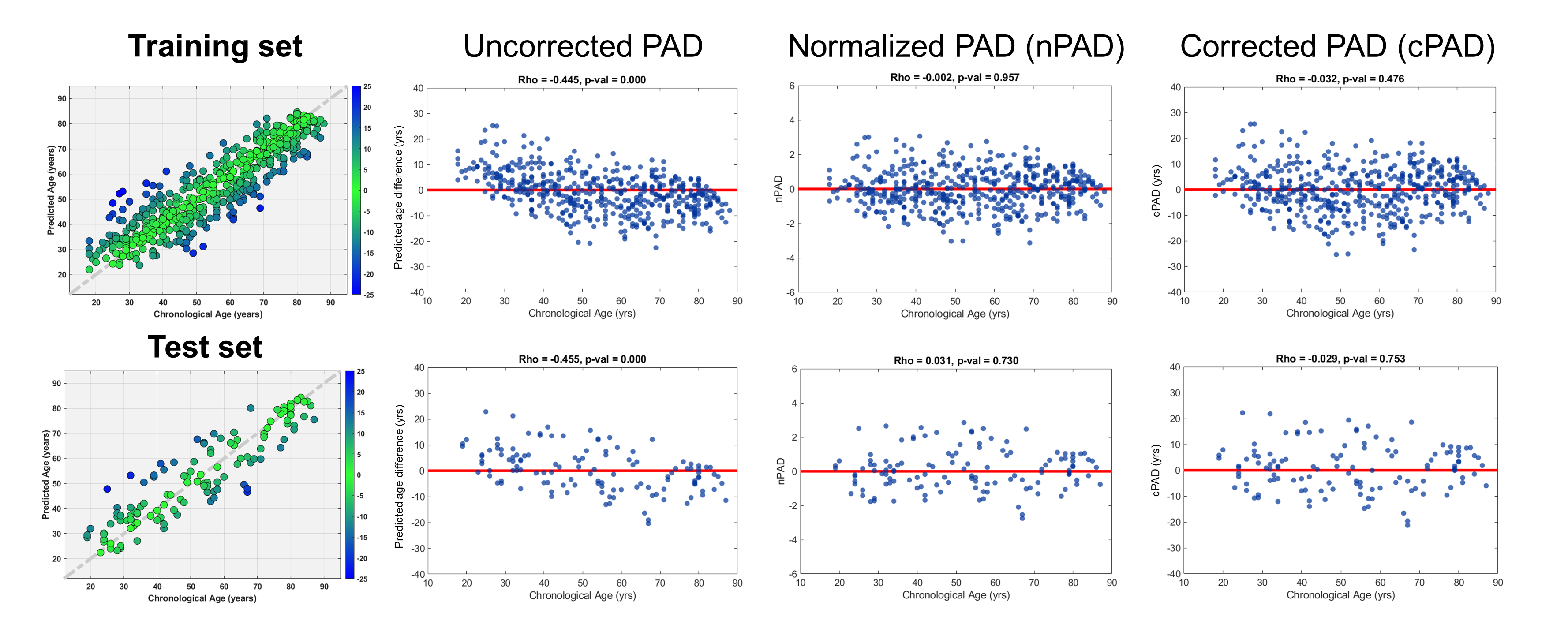


Supplementary Figure 3. The scatter plots of the PAD measures with chronological age. The results were derived from the real data.


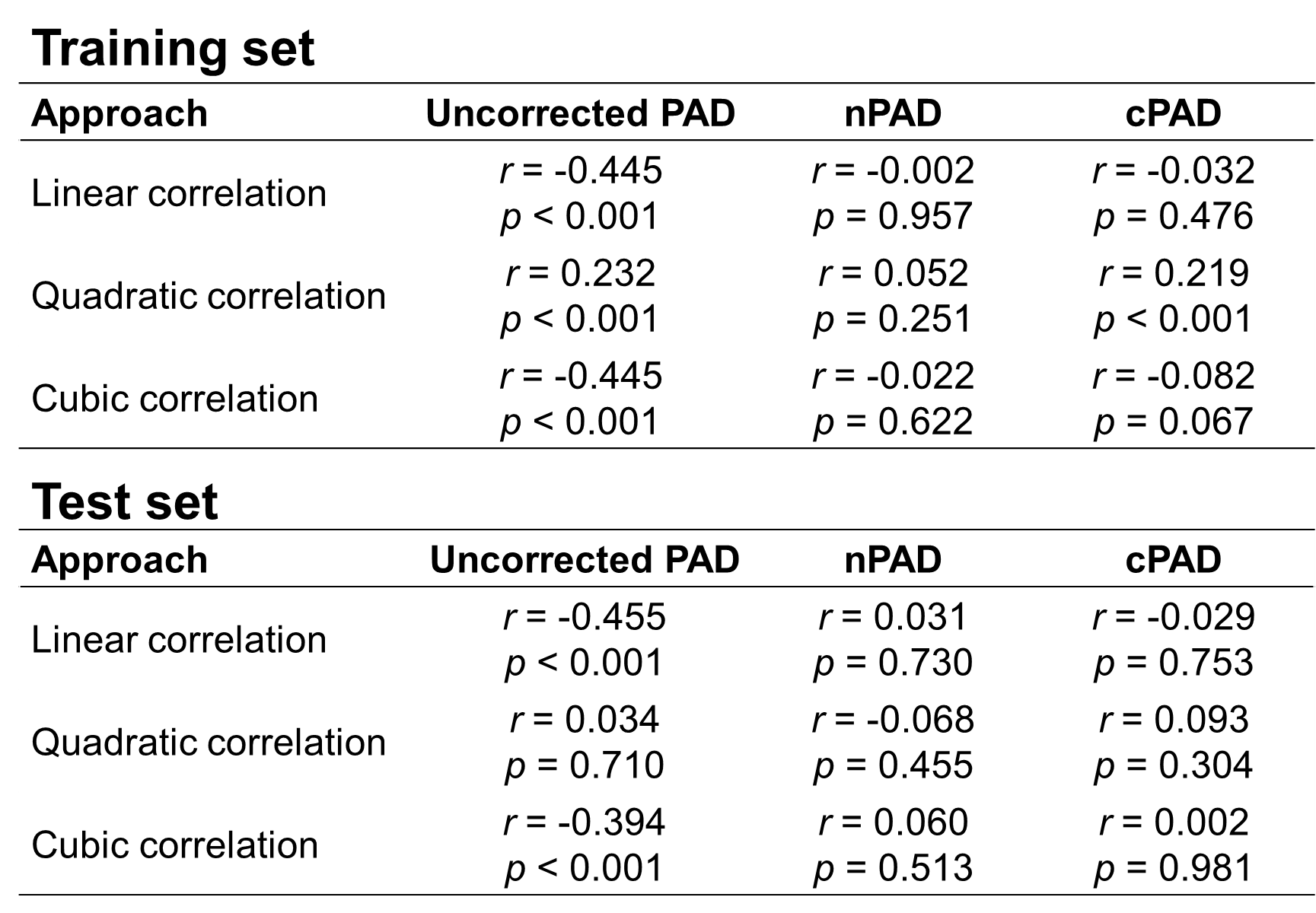


Supplementary Table 2. The linear and non-linear correlations of the PAD measures with chronological age. The results were derived from the real data.

**References**

1. Reese, T.G., Heid, O., Weisskoff, R.M., Wedeen, V.J., 2003. Reduction of eddy-current-induced distortion in diffusion MRI using a twice-refocused spin echo. Magn. Reson. Med. 49, 177–182.

2. Wedeen, V.J., Hagmann, P., Tseng, W.Y., Reese, T.G., Weisskoff, R.M., 2005. Mapping complex tissue architecture with diffusion spectrum magnetic resonance imaging. Magn. Reson. Med. 54, 1377–1386.

3. Kuo, L.W., Chen, J.H., Wedeen, V.J., Tseng, W.Y., 2008. Optimization of diffusion spectrum imaging and q-ball imaging on clinical MRI system. Neuroimage. 41, 7–18.

4. Chen C. L. et al., 2020. Generalization of diffusion magnetic resonance imaging-based brain age prediction model through transfer learning. Neuroimage 217:116831.

5. Huang, G., Liu, Z., Van Der Maaten, L., Weinberger, K.Q., 2017. Densely connected convolutional networks, in Proceedings of the IEEE Conference on Computer Vision and Pattern Recognition. pp. 2261–2269.

6. Chen, C. L. et al., 2019. Premature white matter aging in patients with right mesial temporal lobe epilepsy: A machine learning approach based on diffusion MRI data. Neuroimage Clin 24, 102033, doi:10.1016/j.nicl.2019.102033.

7. Dietrich, O., Raya, J.G., Reeder, S.B., Reiser, M.F., Schoenberg, S.O., 2007. Measurement of signal-to-noise ratios in MR images: influence of multichannel coils, parallel imaging, and reconstruction filters. J. Magn. Reson. Imaging. 26, 375–385.

8. Ashburner, J., Barnes, G., Chen, C., Daunizeau, J., Flandin, G., Friston, K., Kiebel, S., Kilner, J., Litvak, V., Moran, R., 2014. SPM12 manual. Wellcome Trust Centre for Neuroimaging, London, UK, 2464.

9. Ashburner, J., Friston, K.J., 2011. Diffeomorphic registration using geodesic shooting and Gauss-Newton optimisation. Neuroimage. 55, 954–967.

10. Shattuck, D.W., Mirza, M., Adisetiyo, V., Hojatkashani, C., Salamon, G., Narr, K.L., Poldrack, R.A., Bilder, R.M., Toga, A.W., 2008. Construction of a 3D probabilistic atlas of human cortical structures. Neuroimage 39, 1064-1080.

11. Dahnke, R., Ziegler, G., Gaser, C., 2012. Local adaptive segmentation. Beijing. HBM. Available online at: http://dbm. neuro. uni-jena. de/HBM2012/HBM2012-Dahnke02. pdf.

12. Desikan, R.S., Ségonne, F., Fischl, B., Quinn, B.T., Dickerson, B.C., Blacker, D., Buckner, R.L., Dale, A.M., Maguire, R.P., Hyman, B.T., 2006. An automated labeling system for subdividing the human cerebral cortex on MRI scans into gyral based regions of interest. Neuroimage 31, 968-980.

13. Chen, Y.J., Lo, Y.C., Hsu, Y.C., Fan, C.C., Hwang, T.J., Liu, C.M., et al., 2015. Automatic whole brain tract-based analysis using predefined tracts in a diffusion spectrum imaging template and an accurate registration strategy. Hum. Brain Mapp. 36, 3441–3458.

14. Hsu, Y.C., Tseng, W.Y., 2018. An efficient regularization method for diffusion MAP-MRI estimation. 2018 ISMRM-ESMRMB Joint Annual Meeting, Paris, France.

15. Ozarslan, E., Koay, C.G., Shepherd, T.M., Komlosh, M.E., Irfanoglu, M.O., Pierpaoli, C., et al., 2013. Mean apparent propagator (MAP) MRI: a novel diffusion imaging method for mapping tissue microstructure. Neuroimage. 78, 16–32.

16. Avram, A.V., Sarlls, J.E., Barnett, A.S., Ozarslan, E., Thomas, C., Irfanoglu, M.O., et al., 2016. Clinical feasibility of using mean apparent propagator (MAP) MRI to characterize brain tissue microstructure. Neuroimage. 127, 422–434.

17. Alexander, A.L., Lee, J.E., Lazar, M., Field, A.S., 2007. Diffusion Tensor Imaging of the Brain. Neurotherapeutics. 4, 316–329.

18. Le Bihan, D., Mangin, J.F., Poupon, C., Clark, C.A., Pappata, S., Molko, N., et al., 2001. Diffusion tensor imaging: concepts and applications. J. Magn. Reson. Imaging. 13, 534–546.

19. Tuch, D.S., 2004. Q-ball imaging. Magn. Reson. Med. 52, 1358–1372.

20. Hsu, Y.C., Lo, Y.C., Chen, Y.J., Wedeen, V.J., Isaac Tseng, W.Y., 2015. NTU-DSI-122: A diffusion spectrum imaging template with high anatomical matching to the ICBM-152 space. Hum. Brain Mapp. 36, 3528–3541.

21. Tzourio-Mazoyer, N., Landeau, B., Papathanassiou, D., Crivello, F., Etard, O., Delcroix, N., et al., 2002. Automated anatomical labeling of activations in SPM using a macroscopic anatomical parcellation of the MNI MRI single-subject brain. Neuroimage. 15, 273–289.

22. Ashburner, J., Friston, K.J., 2000. Voxel-based morphometry—the methods. Neuroimage 11, 805-821.

23. Hsu, Y.-C., Hsu, C.-H., Tseng, W.-Y.I., 2012. A large deformation diffeomorphic metric mapping solution for diffusion spectrum imaging datasets. NeuroImage. 63, 818–834.

24. Smith, S.M., Vidaurre, D., Alfaro-Almagro, F., Nichols, T.E., Miller, K.L., 2019. Estimation of brain age delta from brain imaging. Neuroimage 200, 528-539.

25. de Lange, A.G., Cole, J.H., 2020. Commentary: Correction procedures in brain-age prediction. Neuroimage Clin 26, 102229.

26. Taylor, J.R., Williams, N., Cusack, R., Auer, T., Shafto, M.A., Dixon, M., Tyler, L.K., Henson, R.N., 2017. The Cambridge Centre for Ageing and Neuroscience (Cam-CAN) data repository: Structural and functional MRI, MEG, and cognitive data from a cross-sectional adult lifespan sample. Neuroimage 144, 262-269.

27. Frees, E. W., & Valdez, E. A., 1998. Understanding relationships using copulas. North American Actuarial Journal, 2(1), 1-25
